# Interaction analysis of tobacco leaf microbial community structure and volatiles flavor compounds during cigar stacking fermentation

**DOI:** 10.3389/fmicb.2023.1168122

**Published:** 2023-08-10

**Authors:** Qiaoyin Wu, Zheng Peng, Yong Pan, Liping Liu, Linlin Li, Juan Zhang, Jian Wang

**Affiliations:** ^1^Key Laboratory of Industrial Biotechnology, Ministry of Education, Jiangnan University, Wuxi, China; ^2^School of Biotechnology, Jiangnan University, Wuxi, China; ^3^Science Center for Future Foods, Jiangnan University, Wuxi, China; ^4^China Tobacco Hubei Industrial Co., Ltd, Wuhan, China

**Keywords:** cigar, stacking fermentation, microbial community, volatile flavor compounds, interaction, aroma

## Abstract

**Introduction:**

Cigar stacking fermentation is a key step in tobacco aroma enhancement and miscellaneous gas reduction, which both have a great influence on increasing cigar flavor and improving industrial availability.

**Methods:**

To analyze the effect of cigar tobacco leaf (CTLs) microbial community on volatiles flavor compounds (VFCs), this study used multi-omics technology to reveal the changes in microbial community structure and VFCs of different cigar varieties during stacking fermentation, in addition to exploring the interaction mechanism of microbiome and VFCs.

**Results:**

The results showed that the dominant microbial compositions of different CTL varieties during stacking fermentation were similar, which included *Staphylococcus*, *Corynebacterium 1*, *Aerococcus*, and *Aspergillus*. These dominant microbes mainly affected the microbial community structure and characteristic microorganisms of CTLs through microbial interactions, thereby influencing the transformation of VFCs. Characteristic microorganisms of different CTLs varieties such as *Trichothecium*, *Trichosporon*, *Thioalkalicoccus* and *Jeotgalicoccus*, were found to positively correlate with characteristic VFCs like megastigmatrienone 4, pyrazine, tetramethyl-, geranyl acetone, and 2-undecanone, 6,10-dimethyl-, respectively. This in turn affected the aroma and sensory quality of the CTLs.

**Discussion:**

This study provides theoretical support for the analysis of the mechanism of microorganisms on VFCs and aroma, and development of microbial agents during cigar stacking fermentation.

## 1. Introduction

Cigars are tobacco products rolled from cigar tobacco leaves (CTLs), and are made through a long process of preparation, fermentation, and aging, ultimately creating a unique and complex flavor (Krusemann et al., 2018; Spatarella et al., 2018). Cigars have various aromas such as nutty, flowery, fruity, and chocolate, and the production of these complex aromas involves the transformation of many substances related to the microbial community and enzymes in CTLs (Gupta, 2015; Krusemann et al., 2020). The content of macromolecular substances and their related enzyme activity change significantly during the growth and agricultural fermentation stages of CTLs (Banozic et al., 2020). Starch, cellulose, and pectin accumulated during CTLs growth and maturation processes are then gradually degraded during the agricultural fermentation stage and subsequently converted into the aroma precursors and VFCs of CTLs (Zhang et al., 2021). At this point, the primary aroma of CTLs has been formed, although the smoke gas remains relatively rough, and miscellaneous gas, bitterness, and other bad flavors need to be further mellowed, in addition to reducing irritation through stacking fermentation to further enrich the aroma of CTLs and improve their quality (Liu F. F. et al., 2022). Stacking fermentation is the industrial fermentation stage in the production process of cigars, and compared with the rapid degradation of macromolecular substances in the growth and agricultural fermentation process, stacking fermentation primarily transforms small molecular substances and VFCs. The content of VFCs changes with the function of microorganisms and related enzymes, although there is little variation in type, and to achieve the effect of increasing aroma, reducing miscellaneous gas, and mellowing the smoke gas (Liu F. F. et al., 2022; Liu T. et al., 2022; Zheng et al., 2022a).

In recent years, the study of aroma type, microbial community composition, and VFCs in cigar stacking fermentation has increased (Liu F. F. et al., 2022; Zheng et al., 2022a), yet the evolution of the microbiome structure during stacking fermentation and its mechanism for characteristic aroma formation remain poorly understood. Therefore, it is necessary to use high-throughput sequencing technology to study the changes in microbial community composition during stacking fermentation, since this is conducive to the mining of functional microorganisms and the analysis of the evolution mechanism of microbial communities, and to further improve the level to which the industrial fermentation production process can be controlled. This is very important for improving the production and process standards related to cigar fermentation (Zhang et al., 2020; Wu et al., 2021; Zhou T. et al., 2021; Zheng et al., 2022b). In this study, different varieties of high-quality CTLs were used as the research objects, whilst the changes in microbial community composition and volatile changes during stacking fermentation were dynamically analyzed. The correlation network between microorganisms and volatile aroma was first established, with the changes in microbial community structure during stacking fermentation and the influence of this on VFCs and aroma subsequently analyzed. This study provides guidance for analyzing the mechanism of stacking fermentation and the excavation of functional microorganisms involved in this.

## 2. Materials and methods

### 2.1. Sample collection

Eight high-quality CTL samples were collected from China Tobacco Hubei Industry Co., Ltd., including different varieties of CX14, DX4, CIOLLO 98, N-Jalap HABANA, HVA, BESUKI, MATA FINA, and E-HABANO 2000. The entire stacking fermentation process consists of a total of five nodes, for every 7 d of fermentation, or temperature reached 45°C for sampling and re-stacking. Five nodes were named, as raw materials (T0, 0–7 d), pre-fermentation (T1, 7–14 d), mid-fermentation (T2, 14–21 d), post-fermentation (T3, 21–28 d), and end of fermentation (T4, 28–35 d). Further information regarding these samples is presented in Supplementary Table S1.

### 2.2. CTLs sampling

CTL samples of 5 g were added to a sterile pre-chilled mortar, before including liquid nitrogen, and this mixture was then ground into a powder. The CTL powder was put into a 500 mL shaker flask containing 100 mL of sterile normal saline and was subsequently treated at 10°C and 220 rpm for 2–3 h. After the elution was filtered, CTL powder was removed by filtering through four layers of gauze, before the filtrate was centrifuged at 4°C and 7,000 rpm for 20 min. The precipitate was then collected to represent the total microorganism content of the CTLs. These collected microbial cells were extracted using the DNeasy PowerSoil Pro Kit to show CTL microbial genomic DNA. The extracted DNA samples were then amplified through PCR using the primers ITS1F (5’-GGAAGTAAAAGTCGTAACAAGG-3′)-ITS2R (5’-GCTGCGTTCTTCATCGATGC-3′) and 515F (5’-GTGCC AGCMGCCGCGGTAA-3′)-907R (5’-CCGTCAATTCMTTTRAG TTT-3′) for fungi and bacteria, respectively. The amplification products were then electrophoresed on 1% agarose gel, with the corresponding products being recovered for further analysis.

### 2.3. Amplicon sequencing

Here, the Illumina platform was used to sequence the DNA fragments of the microbial community using the two-ended sequencing method, silva_132 and unite_8, whilst the sequence analysis method used was DADA2 (Callahan et al., 2016).

### 2.4. Profiling of VFCs by HS-SPME-GC-MS during stacking fermentation

Using the DB-5MS column with headspace solid-phase microextraction (50/30 DVB/CAR/PDMS fiber), the helium flow rate was constant at 1.0 mL/min. The temperature procedure was as follows: at the beginning of injection, the column temperature was 40°C (hold for 2 min) and then increased to 250°C (hold for 5 min) at a rate of 10°C/min. The ion source temperature was maintained at 210°C and the transfer line temperature was maintained at 280°C. The system operated in EI mode with an EI voltage of 70 eV. Full scan mode used a mass scan range of 33–400 m/z with an acquisition rate of 10 scans per second (Zheng et al., 2022a). Compared with chemical standards in National Institute of Standards and Technology spectral library WILEY 8.0 and NIST14, VFCs with similarity above 800 and flavor characteristics were selected for analysis, and the relative content was calculated by the area normalization method (Wu et al., 2022).

### 2.5. Sensory quality analysis

The sensory evaluation of CTL samples was carried out by China Tobacco Hubei Industry Co., Ltd. The indexes of the samples were scored 1–5 points, whilst the non-existent aroma and bad flavors were scored 0 points. Aromas were divided into woody, bean, flower, honey sweet, mellow sweet, burn sweet, light sweet, pollen, milk, hay, roasted, resin, fruity, herb, nutty, leather, coffee and pepper. Bad flavor was divided into green miscellaneous gas, burning, woody gas, earthy, regional gas, and protein.

### 2.6. Data analysis

The correlations between microorganisms and VFCs, and between VFCs and aroma were calculated using the psych package for R software (version 4.2.2) (Yu S. et al., 2021). Furthermore, the pheatmap package was used to create a heatmap of the VFCs, whilst Gephi (version 0.9.4) and Cytoscape (version 3.9.1) software were used for the network of interactions (Shannon et al., 2003), the (Partial Least Squares Discriminant Analysis) PLS-DA model of SIMCA (version 14.1) was used to screen CTLs characteristic VFCs (Ruiz-Perez et al., 2020), and Origin 2021 and Excel 2021 software were used for data processing and analysis.

## 3. Results

### 3.1. Microbial community changes during stacking fermentation

**Figure 1:**
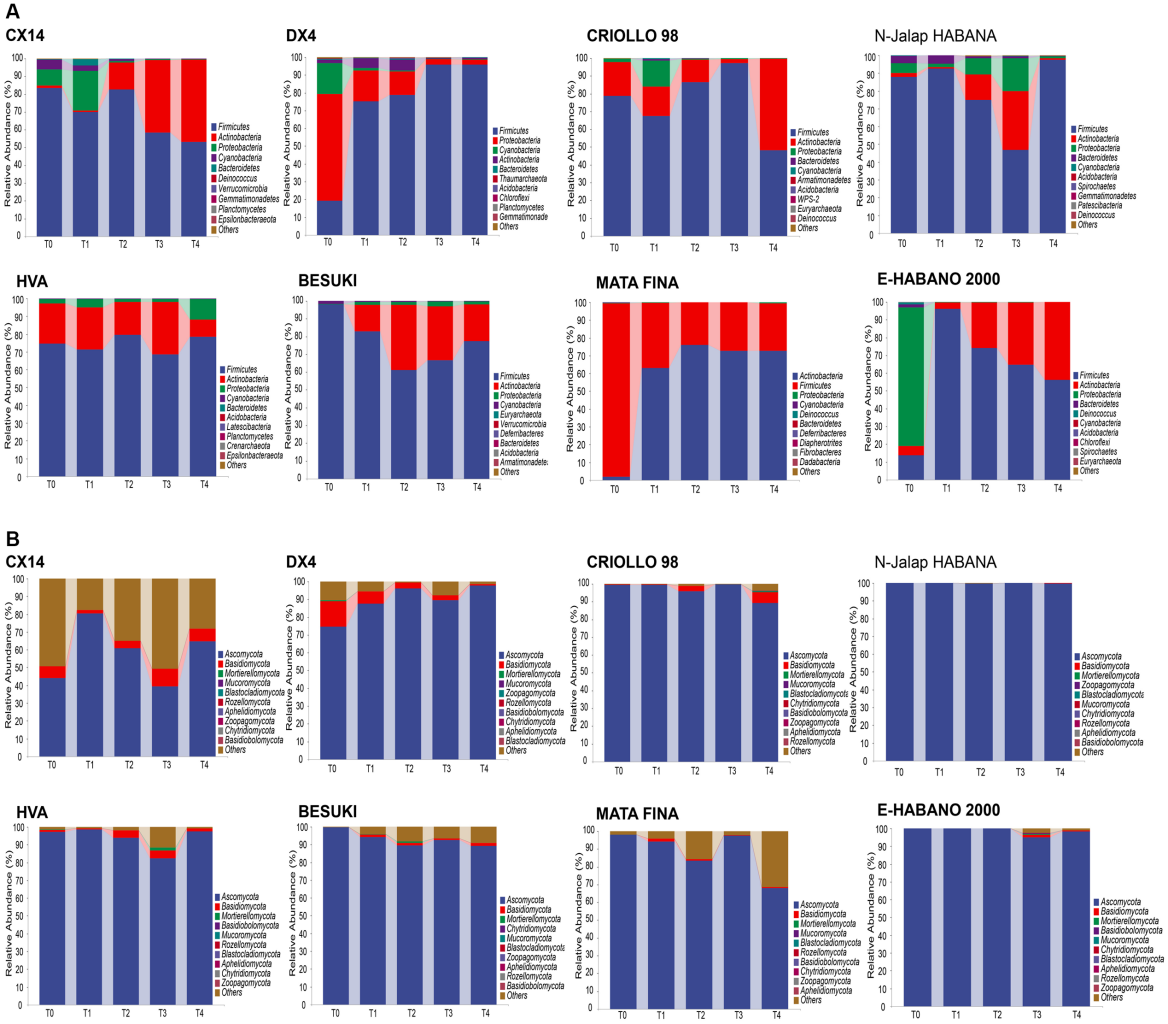
Microbial community changes of CTLs from different varieties at the phylum level. **(A)** Composition of bacterial community during stacking fermentation. **(B)** Composition of fungal community during stacking fermentation.

The changes in microbiota structure during cigar stacking fermentation were analyzed by 16S and ITS amplicon sequencing, the bacterial and fungal phylum with relative abundances in the top 10 were selected for subsequent analysis, as shown in Figure 1. *Firmicutes*, *Actinobacteria* and *Proteobacteria* were the dominant bacterial phylum in the different varieties of CTLs. During stacking fermentation, the relative abundance of *Firmicutes* was gradually increased in DX4 CTLs; *Actinobacteria* increased to 46.30%, 51.70%, 20.63%, 72.70% and 43.59% in CX14, CRIOLLO 98, BESUKI, MATA FINA and E-HABANO 2000 CTLs, respectively. In HVA and N-Jalap HABANA CTLs, relative abundance of *Firmicutes* had changed, with 78.82% and 97.38% at the end of fermentation. *Ascomycota* was the primarily dominant fungi at the phylum level during stacking fermentation, the relative abundance of different CTLs varieties from 40.54% to 99.46%.

**Figure 2:**
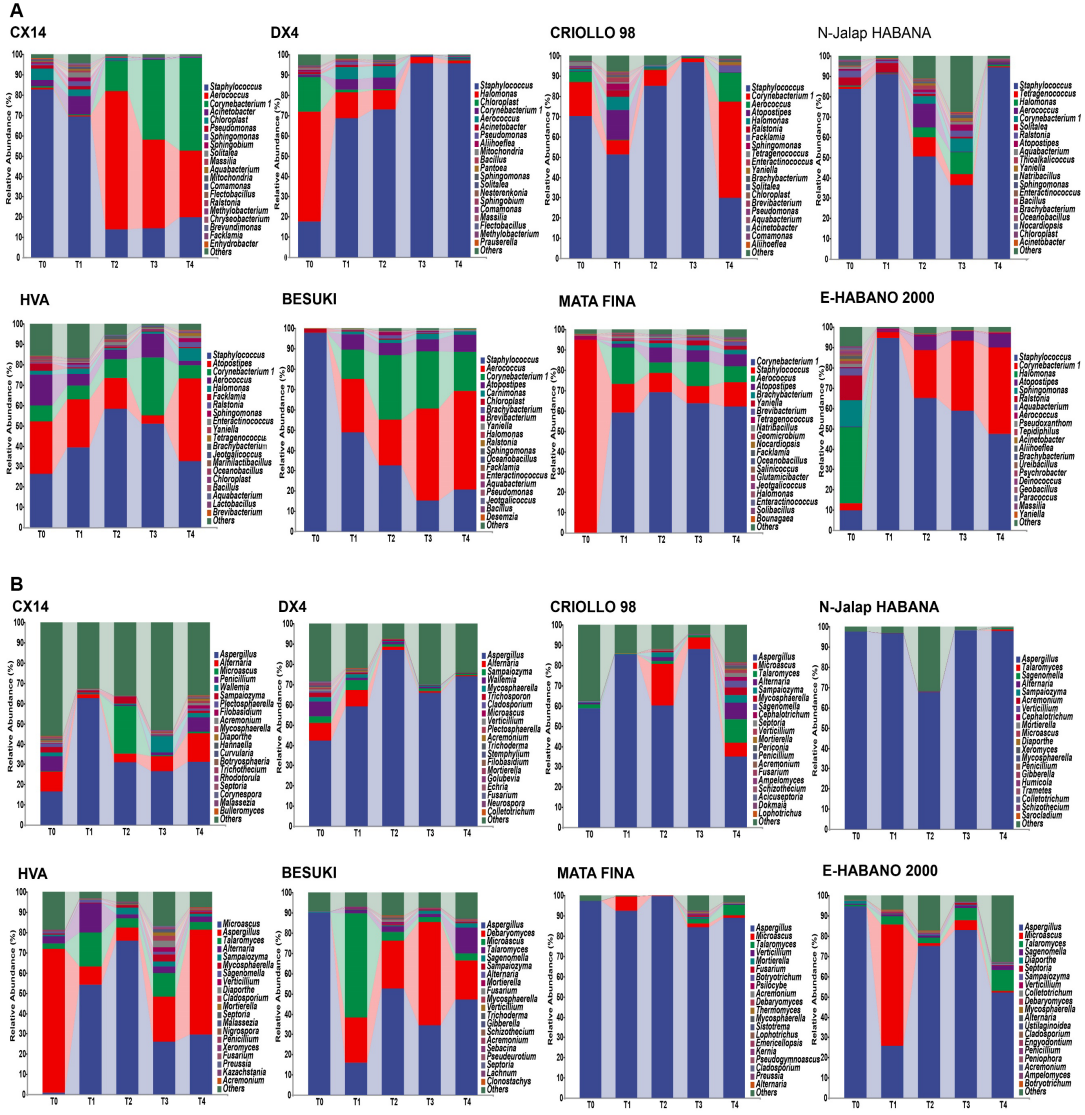
Microbial community changes of CTLs from different varieties at the genus level. **(A)** Composition of bacterial community during stacking fermentation. **(B)** Composition of fungal community during stacking fermentation.

The bacterial and fungal genera with relative abundances in the top 20 were selected for subsequent analysis, as shown in Figure 2. The results showed that the compositions of the dominant bacterial genera in the different varieties of CTLs were similar. The three dominant bacterial genera, *Staphylococcus*, *Corynebacterium 1* and *Aerococcus*, were the main components of the bacterial communities in different CTL varieties. The total relative abundance of all samples was between 67.15% and 99.10%; however, the relative abundance changes during stacking fermentation differed. The relative abundances of *Corynebacterium 1* and *Aerococcus* gradually increased from the CX14, BESUKI, and MATA FINA CTLs, while the sum of the relative abundances of *Corynebacterium 1* and *Aerococcus* at the end of fermentation were 79.59%, 67.68%, and 72.30%, respectively. The CTLs from DX4 and N-Jalap HABANA had *Staphylococcus* as the dominant bacteria in the stacking fermentation process, with its relative abundance exceeding 90% at the end of fermentation. Furthermore, the CTLs from CRIOLLO 98, HVA, and E-HABANO 2000 showed *Staphylococcus* as the main dominant bacteria, although the relative abundance of *Staphylococcus* gradually decreased, whilst the relative abundances of *Halomonas*, *Atopostipes*, *Corynebacterium 1*, and other genera showed a slight increase. The high relative abundance of dominant bacteria *Staphylococcus*, *Corynebacterium 1* and *Aerococcus* in stacking fermentation might be due to the decrease of tobacco leaf water content and the increase of pH with the fermentation process, and the strains had better alkali and salt resistance, making them in a dominant position of staking fermentation (Di Giacomo et al., 2007; Li et al., 2020).

During stacking fermentation, the fungal community with *Aspergillus* as the dominant fungal genus showed the highest proportion of relative abundance among different varieties of CTL fungi, whilst the relative abundance of *Aspergillus* in different CTLs varied greatly. Among them, CX14, CRIOLLO 98, HVA, BESUKI, and E-HABANO 2000 CTLs *Aspergillus* decreased during the fermentation process, with the community composition being richer at the end of fermentation. While *Aspergillus* was the most dominant fungus in the CTLs from DX4, N-Jalap HABANA, and MATA during stacking fermentation, at the end of fermentation, the relative abundances of *Aspergillus* were 73.98%, 97.83%, and 88.88%, respectively. *Aspergillus*, the dominant fungus genus of stacked fermentation, is a common high-abundance fungus genus in solid-state fermentation, which can produce a variety of enzymes such as protease and amylase, and has the functions of increasing flavor and regulating flora (Zhou J. et al., 2021; Zhu et al., 2022).

The above results showed that the dominant microorganisms of cigar stacking fermentation were *Staphylococcus*, *Corynebacterium 1*, *Aerococcus* and *Aspergillus*, which were similar to the results of the research results of Zheng L. L. et al. (2022), under the influence of the chemical composition of tobacco leaves, tobacco endophytes and fermentation environment, the relative abundance of dominant microorganisms of different varieties changes were different, which further affected the changes of microbial communities of different CTLs varieties.

### 3.2. Characteristic microorganisms from different varieties of CTLs

**Figure 3:**
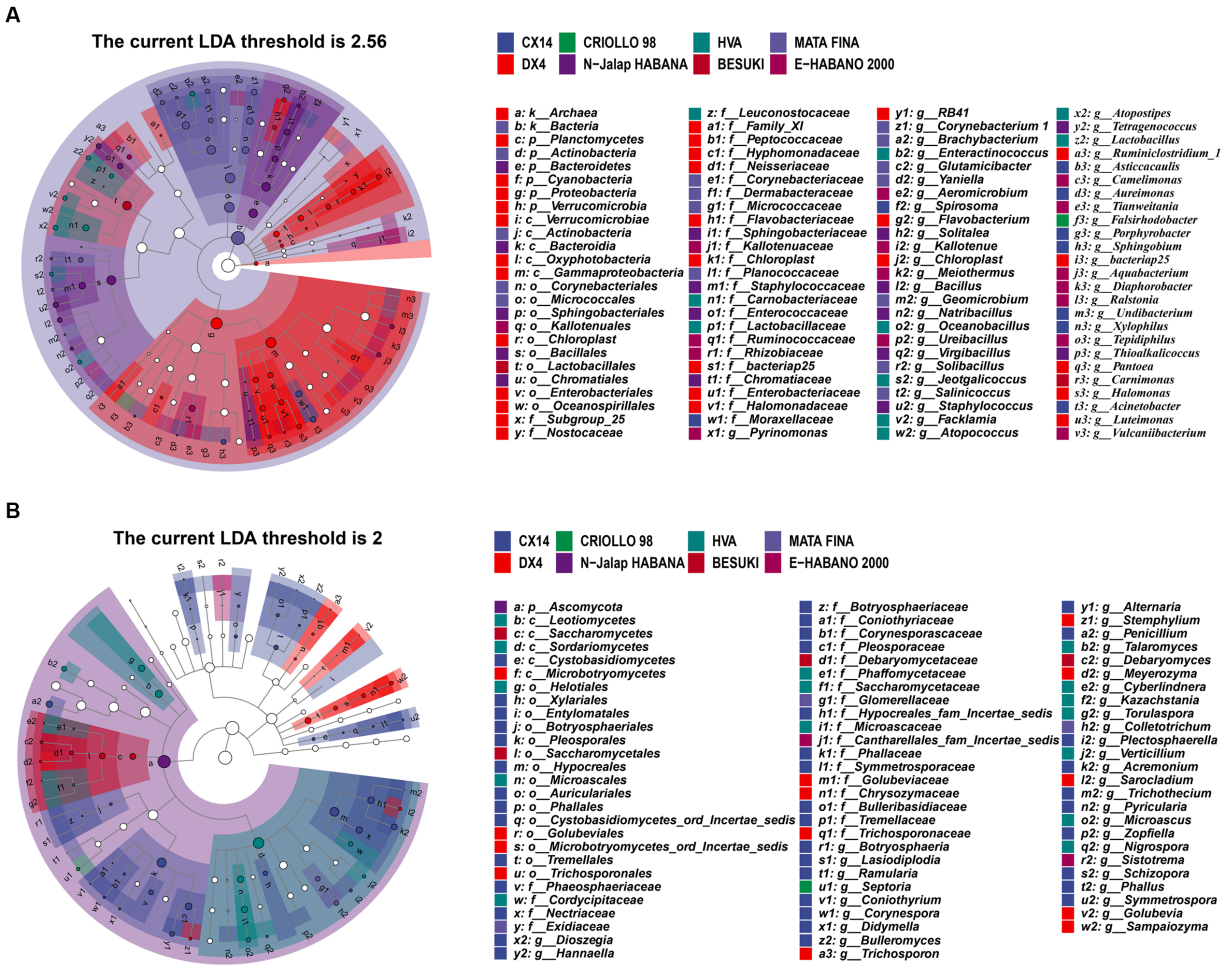
Characteristic microorganisms from different varieties of CTLs. **(A)** Characteristic bacterium. **(B)** Characteristic fungus.

The results showed that the dominant genera and community structures of different samples affected the metabolism of microbial communities, whilst the characteristic microorganisms were significantly correlated with the characteristic VFCs. For example, *Staphylococcus* and *Bacillus*, were found to have been positively correlated with sclareolide and isophorone, respectively (Liu et al., 2021; Liu T. et al., 2022; Yan et al., 2022; Zheng T. F. et al., 2022), therefore the characteristic microorganisms of different CTL varieties may have had an important influence on the composition of the characteristic aroma. Linear discriminant analysis effect size (LEfSe) was used to identify the characteristic microorganisms from different varieties of CTLs (LDA score > 2), and the results of this are shown in Figure 3. Among these, DX4 CTLs had the largest number of characteristic bacterial genera, whilst BESUKI CTLs had the fewest characteristic bacterial genera. Additionally, CX14 CTLs showed the largest number of characteristic fungal genera, whilst N-Jalap HABANA CTLs had the fewest characteristic fungal genera.

The different microbial genera of different varieties of tobacco leaves are as follows: CX14 CLTs *Sphingobium*, *Pseudomonas*, *Acinetobacter*, *Penicillium*, *Diaporthe*, etc.; *Halomonas*, *Trichosporon*, etc. in DX4 CTLs; *Falsirhodobacter*, *Septoria*, etc. in CRIOLLO 98 CTLs; N-Jalap HABANA CTLs of *Sphingobacterium*, *Solitalea*, *Thioalkalicoccus*, etc.; *Enteractinococcus*, *Lactobacillus*, *Jeotgalicoccus*, *Talaromyces*, etc. in HVA CTLs; *Carnimonas*, *Debaryomyces*, etc. in BESUKI CTLs; *Brachybacterium*, *Brevibacterium*, *Glutamicibacter*, *Yaniella*, etc. in MATA FINA; E-HABANO 2000 CTLs of *Ralstonia*, *Deinococcus*, *Sphingomonas*, etc. The different microorganisms of CTLs, such as *Pseudomonas*, *Sphingomonas*, *Acinetobacter*, *Lactobacillus*, *Trichothecium*, *Nigrospora*, were common genus in tobacco endophytes. Within strong salt tolerance, they could better adapt to the alkaline environment of CTLs, and had the effects of promoting growth, resistance to diseases and pests, and pollutant degradation. They had great economic value and application development potential (Li et al., 2019; Gushgari-Doyle et al., 2021; Gibot-Leclerc et al., 2022).

### 3.3. Interaction relationship between microbial community

**Figure 4:**
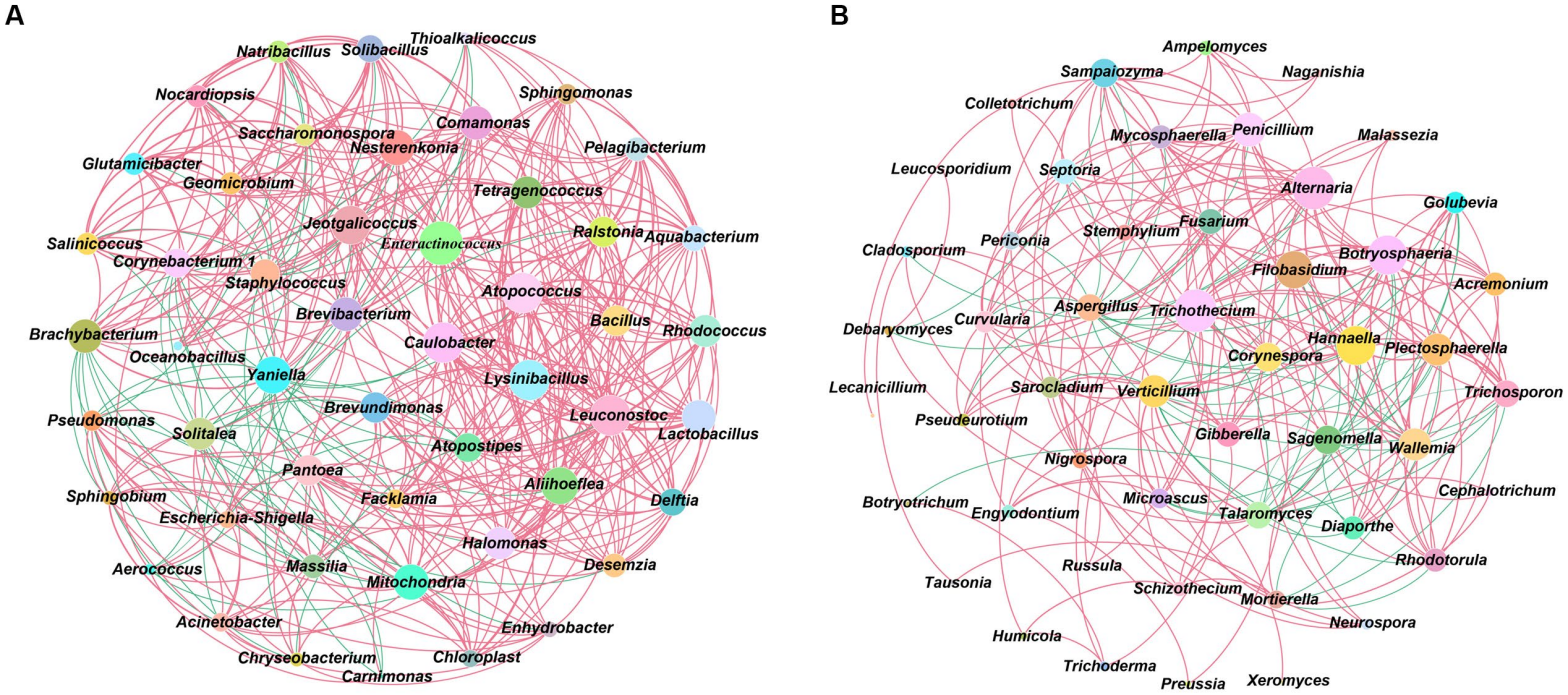
Microbial community interaction networks. **(A)** Interaction network between CTLs bacterial communities. **(B)** Interaction network between CTLs fungal communities. Red represents positive correlation and blue represents negative correlation.

Using Spearman to analyze the interaction relationship between CTL microbial communities, *ρ* < 0.05, *r* > 0.6 were defined as significant positive correlations, whilst *ρ* < 0.05, *r* < −0.6 were defined as significant negative correlations, with the results being shown in Figure 4. During stacking fermentation, among the dominant bacteria, *Staphylococcus* were negatively correlated with *Corynebacterium 1* and *Aerococcus*, whilst *Corynebacterium 1* and *Aerococcus* interacted positively. Dominant bacterial genera from different CTL varieties were significantly related to characteristic microorganisms, for example, *Corynebacterium 1* was positively correlated with *Facklamia*, *Jeotgalicoccus*, *Brachybacterium*, *Geomicrobium*, *Glutamicibacter*, *Yaniella*, *Salinicoccus*, *Solibacillus*, and *Natribacillus*. Additionally, *Aerococcus* was positively correlated with *Carnimonas*, *Facklamia*, *Yaniella*, whilst *Staphylococcus* was shown to have been positively correlated with *Halomonas* and *Solitalea*.

During stacking fermentation, the dominant bacterial genera were found to have affected the growth of the corresponding characteristic bacterial genera, such as *Aerococcus* and *Corynebacterium 1*, which in turn increased in the pre-fermentation period. This promoted the proliferation of *Sphingobium*, *Aquabacterium*, *Halomonas*, etc., and decreased when *Staphylococcus* increased during the middle and late fermentation stages.

*Aspergillus*, the dominant fungi of CTLs, was negatively correlated with characteristic fungal genera, including *Alternaria*, *Curvularia*, *Fusarium*, *Gibberella*, *Mycosphaerella*, *Plectosphaerella*, *Pseudeurotium*, *Penicillium*, *Sampaiozyma*, *Septoria*, and *Trichothecium*. During stacking fermentation, *Aspergillus* inhibits the growth of characteristic fungi, such as *Alternaria*, *Trichosporon*, and *Mycosphaerella* which was opposite to the change observed in *Aspergillus*. The abundance of *Aspergillus* was found to have been higher during the early stage of fermentation, whilst the characteristic fungus was then inhibited from growing and increased during the middle and late stages of fermentation with a decrease in *Aspergillus*. The above results showed that the abundance and interactions of dominant microbes during stacking fermentation affected the changes in characteristic microorganisms in the microbial community and subsequently influenced the microbiota structure of CTLs during the stacking fermentation process.

Characteristic bacteria such as *Aquabacterium*, *Brachybacterium*, *Tetragenococcus*, *Ralstonia*, *Enteractinococcus*, *Jeotgalicoccus*, *Lactobacillus*, *Bacillus*, and *Yaniella* all interacted in a positive direction, whilst characteristic fungi such as *Sampaiozyma*, *Wallemia*, *Penicillium*, *Trichosporon*, *Nigrospora*, *Septoria*, and *Plectosphaerella* also all interacted in a positive direction. The interactions between characteristic microorganisms had an important impact on the evolution of microbiota structure during stacking fermentation (Wang et al., 2019). Zhou J. et al. (2021) defined *Mycosphaerella*, *Plectosphaerella*, *Bacillus*, *Methylobacterium* and *Fusarium* as symbiotic flora microorganisms that have a greater impact on the structure and function of microbial flora. Wang et al. (2019) defined *Lactobacillus, Saccharomyces*, *Pichia*, *Geotrichum* and *Candida* with aroma production ability as the symbiotic microorganisms of simulated artificial fermentation flora in the liquor mixing system. This were similar to the natural fermentation system in the direction of succession in the artificially constructed fermentation system. Therefore, the abundance and interaction between microorganisms of the dominant genus during stacking fermentation affect the changes of characteristic microorganisms in the community, and as a symbiotic group in the stacking fermentation microbial community, the characteristic microorganisms can affect the succession of tobacco leaf flora structure during stacking fermentation.

### 3.4. Changes of VFCs in the stacking fermentation

**Figure 5:**
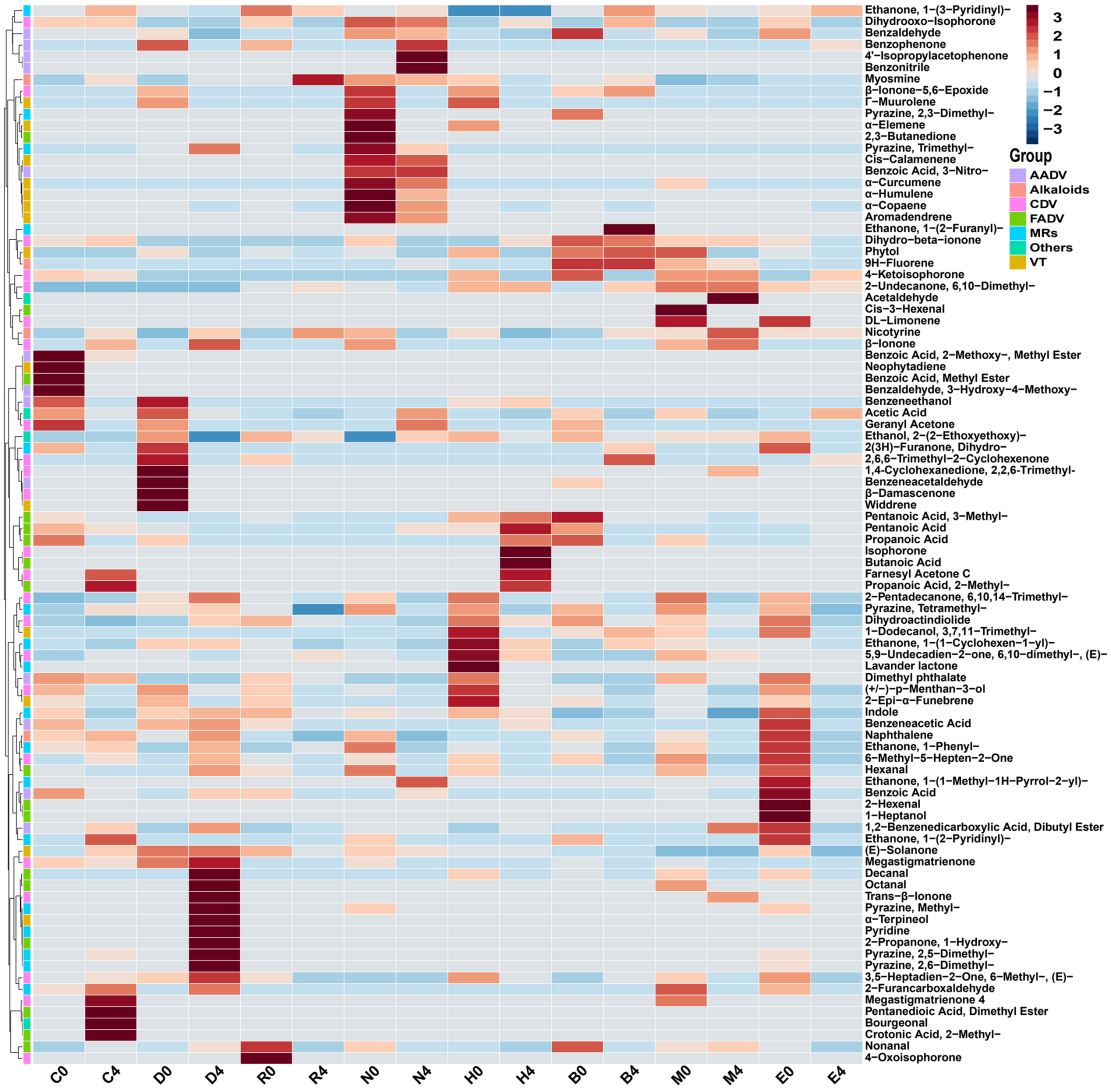
Cluster analysis of VFCs from different varieties of CTLs between raw materials and the end of fermentation. AADV, aromatic amino acid degradation products; Alkaloids, nicotinic degradation products; CDV, carotenoid degradation products; FADV, fatty acid degradation products; MRs, Maillard reaction products; VT, terpenoids; Others, Other VFCs.

The results showed that the types of VFCs increased during stacking fermentation with the VFCs detected including 2-undecanone, 6,10-dimethyl-, geranyl acetone, β-ionone, phytone, farnesyl acetone, ethanone, 1-(3-pyridinyl)-, myosmine, and nicotyrine (Liu F. F. et al., 2022). However, the transformation mechanism of stacking fermentation on CTL aroma and sensory quality remained unclear. The results are shown in Figure 5, with the VFCs at the end of fermentation for different CTLs and the VFCs of CTLs between raw materials and the end of fermentation being found to have been quite different. During stacking fermentation, carotenoid degradation products, Maillard reaction products, and nicotinic degradation products increased, which was consistent with the past results found by Liu’s study, indicating that these three types of substances were the key VFCs for the quality improvement of CTLs (Liu F. F. et al., 2022).

**Figure 6:**
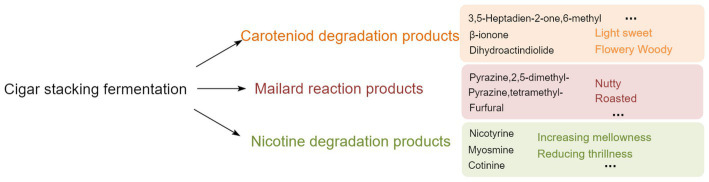
Effects from VFCs of stacked fermented CTLs on the aroma and quality.

In addition to β-ionone, dihydroactinidiolide, 6-methyl-3,5-heptadien-2-one, geranyl acetone, and isophorone, the carotenoid degradation products in different varieties of CTLs also contained β-ionone-5,6-epoxide, menthol, and α-terpineol, among other aroma intermediates. Carotenoid degradation products are important components of tobacco aroma, the threshold is low, and their type and content both have an important impact on improving tobacco aroma and mellowness (Popova et al., 2019; Liang et al., 2021). For example, β-ionone is an aroma ingredient of CTLs flowery and woody aroma; dihydroactinidiolide has a sweet and woody aroma, which could mellow the smoke gas; 6-methyl-3,5-heptadien-2-one and geranyl acetone have a rosy, leafy, and fruity aroma (Liang et al., 2021). The content of Maillard reaction products, such as pyrazine, 2,5-dimethyl-, pyrazine, 2,6-dimethyl-, pyrazine, tetramethyl-, furfural, 2-propanone, 1-hydroxy-, ethanone, 1-(3-pyridinyl)-, and indole, all increased during stacking fermentation. Maillard reaction products are mainly generated by carbohydrates and amino acids via the production of dicarbonyl compounds and amino ketones, primarily creating nutty, roasted, chocolate, and other aromas for CTLs (Mortzfeld et al., 2020). Nicotine, the most abundant and important alkaloid in CTLs, is irritating and bitter when its content is too high, whilst there is insufficient smoke and a bland flavor when its content is too low. CTLs can reduce irritation and increase mellowness through degrading nicotine to produce myosmine and nicotine during stacking fermentation (Lin et al., 2016). Yao et al. (2022) inoculated *Saccharomycopsis fibuligera* and *Hanseniaspora uvarum* in cigar core tobacco leaves, the content of nicotine in fermented CTLs was significantly reduced, the content of carotenoid degradation products such as phytone, farnesyl acetone and limonene increased, and the Maillard reaction products such as 3-acetylpyridine, pyridine and furan increased, and the aroma of CTLs has been improved to varying degrees. Therefore, in stacking fermentation, the transformation of carotenoid degradation and Maillard reaction products alongside the degradation of nicotine played a role in mellowing smoke gas and enhancing the aroma of CTLs (see Figure 6).

### 3.5. Characteristic VFCs from different CTLs varieties

**Figure 7:**
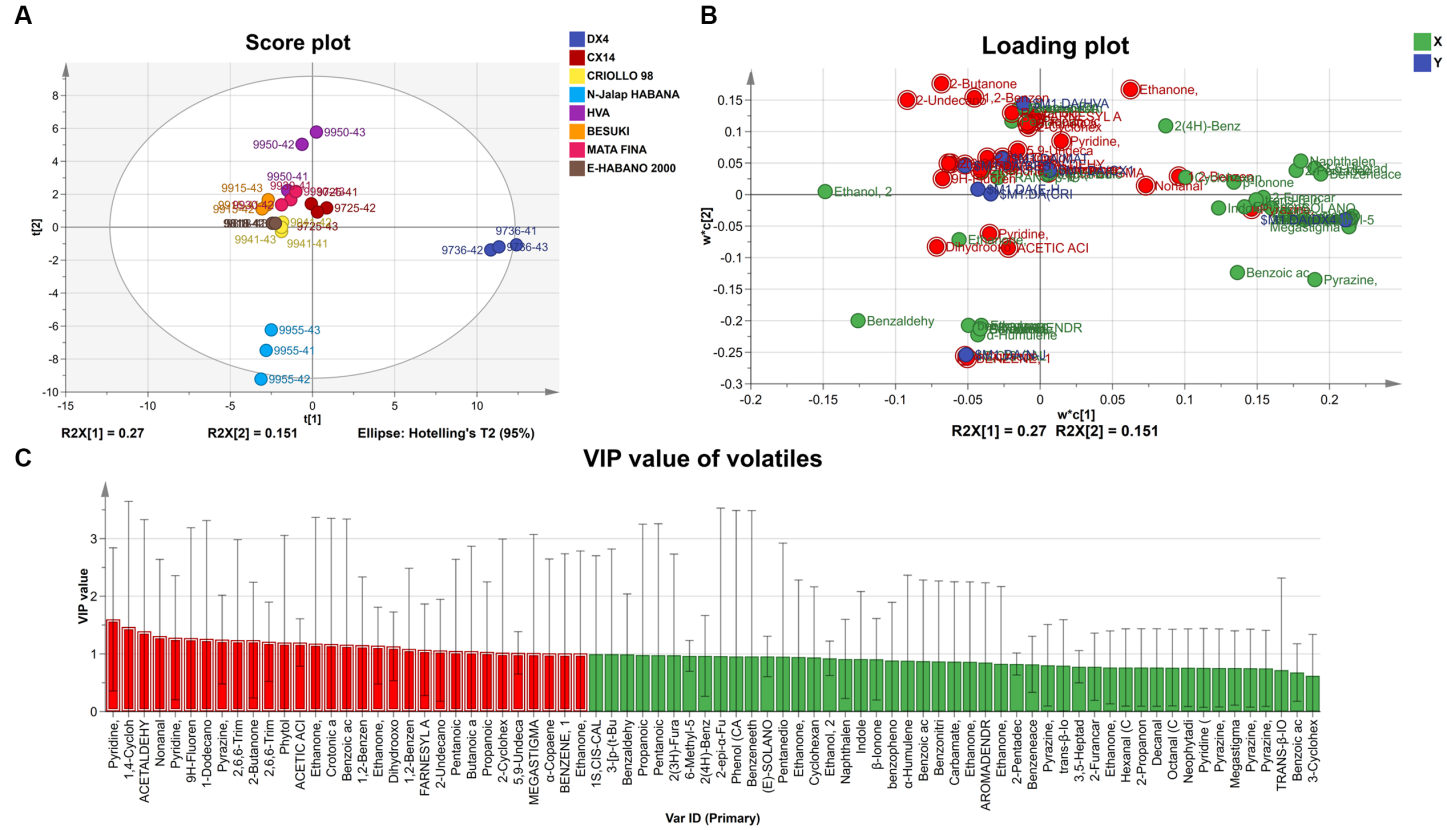
PLS-DA Analysis of VFCs at the end of fermentation. **(A)** PLS-DA score plot of VFCs. **(B)** Loading plot of VFCs. **(C)** VIP value of VFCs.

The characteristic VFCs of different CTL varieties were analyzed using partial least squares discriminant analysis (PLS-DA), and the results are shown in Figure 7. The model Q^2^ value was 0.809, indicating a better model prediction effect. VFCs with a VIP value greater than 1 were defined as characteristic VFCs, which contributed more to the difference between samples. The characteristic VFCs at the end of the fermentation of CTL varieties are shown in Table 1.

**Table 1:** Characteristic VFCs of different varieties of CTLs.

Varieties	Characteristic VFCs
CX14	Ethanone, 1-(2-pyridinyl)-, Tiglic acid, Benzoic acid, 2-methoxy-, methyl ester, Dimethyl phthalate, Megastigmatrienone 4, Bourgeonal
DX4	Nonanal, Pyrazine, tetramethyl-, Ethanone, 1-(1-Cyclohexen-1-yl)-
CRIOLLO 98	Myosmine
N-Jalap HABANA	Acetic Acid, Geranyl Acetone, α-Curcumene
HVA	Farnesyl acetone C, 2-Undecanone, 6,10-dimethyl-, Pentanoic acid, 3-methyl-, Butanoic acid, Propanoic acid, 2-methyl-, Isophorone, α-Copaene
BESUKI	9H-Fluorene, 1-Dodecanol, 3,7,11-Trimethyl-, 2,6,6-Trimethyl-2-Cyclohexenone, Dihydro-beta-ionone, Phytol, Dihydrooxo-isophorone, Ethanone, 1-(2-furanyl)-
MATA FINA	1,4-Cyclohexanedione, 2,2,6-Trimethyl-, Acetaldehyde, Nicotyrine, 1,2-Benzenedicarboxylic acid, dibutyl ester, 4-Oxoisophorone

### 3.6. Correlation analysis between microorganisms-VFCs and VFCs-aroma

**Figure 8:**
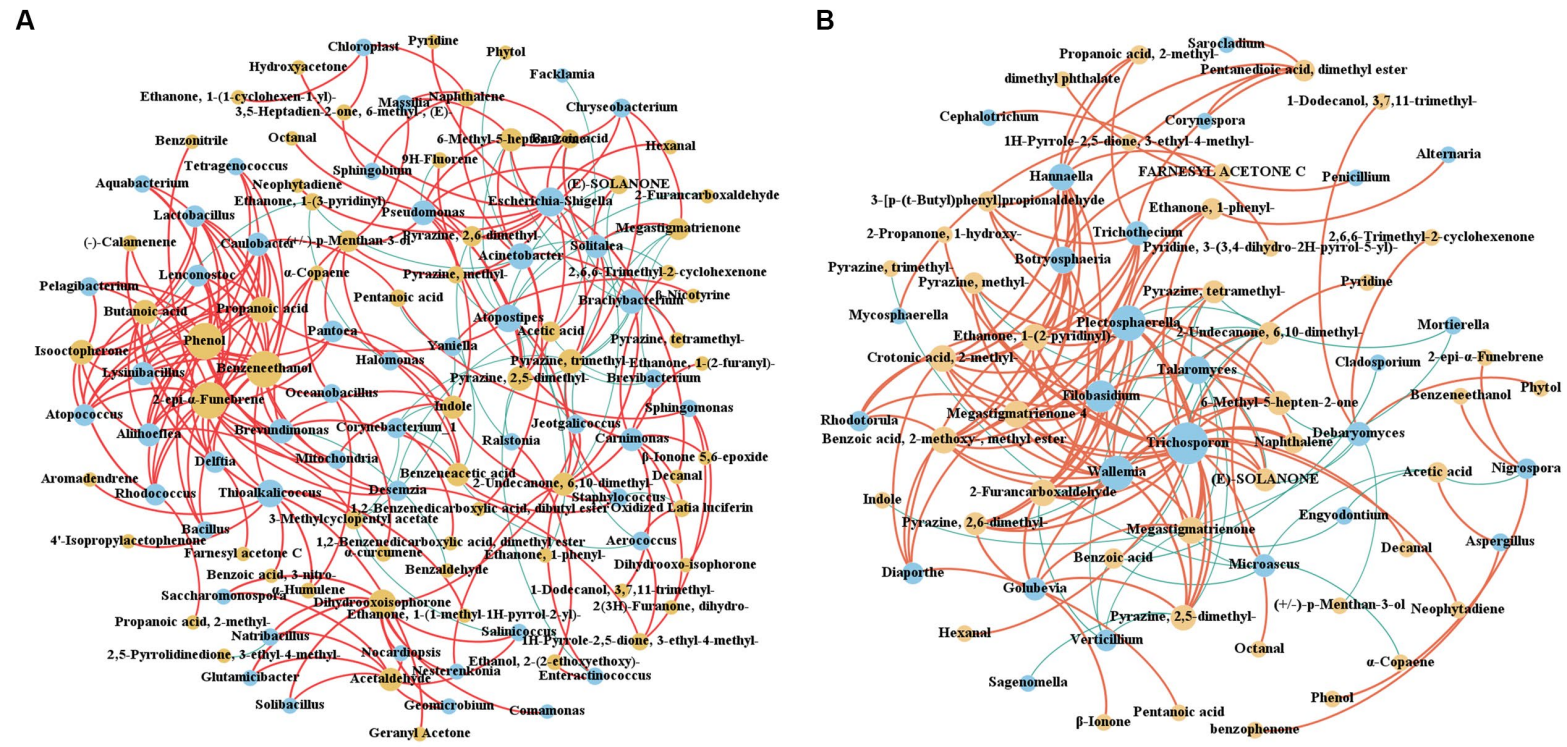
Interaction network between CTLs microorganisms and VFCs. **(A)** Interaction network between bacteria and VFCs. **(B)** Interaction network between fungi and VFCs. Red represents positive correlation and blue represents negative correlation.

The interaction between CTLs microorganisms and VFCs during stacking fermentation was analyzed by Spearman correlation analysis, with *ρ* < 0.05 and *r* > 0.6 being defined as significant positive correlations, and *ρ* < 0.05 and *r* < −0.6 being defined as significant negative correlations, as shown in Figure 8. The correlation between bacteria and VFCs during stacking fermentation was more complex than that between fungi and VFCs whilst also being mainly positively correlated. Characteristic VFCs of different varieties of CTLs were also positively correlated with characteristic microorganisms, such as in CX14 CTLs, *Penicillium* was positively correlated with dimethyl phthalate, whilst *Diaporthe*, *Trichothecium* were positively correlated with ethanone, 1-(2-pyridinyl)-, tigelic acid, megastigmatrienone 4, and bourgeonal. This provides CTLs with a fresh flowery and light sweet aroma. In DX4 CTLs, *Chloroplast* was positively correlated with ethanone, 1-(1-cyclohexen-1-yl)-, and *Trichosporon* was positively correlated with pyrazine, tetramethyl-, which could increase the roasted and burnt sweet aroma of CTLs. Among N-Jalap HABANA CTLs, *Thioalkalicoccus* was positively correlated with geranyl Acetone and α-curcumene, and *Solitalea* was positively correlated with acetic acid, which could increase the light sweet and flowery aroma of CTLs. Furthermore, *Enteractinococcus*, *Jeotgalicoccus*, and *Talaromyces* in the HVA CTLs were positively correlated with 2-undecanone, 6,10-dimethyl-, and *Lactobacillus*, whilst *Atopococcus* was positively correlated with butanoic acid and isophorone, which could increase the fruity and mint aroma. *Carnimonas* and *Debaryomyces* in BESUKI CTLs were also positively correlated with 1-dodecanol, 3,7,11-trimethyl- and dihydro-beta-ionone, which could increase the flowery and hay aroma. Additionally, *Yaniella* in MATA FINA CTLs was positively correlated with nicotine, when *Salinicoccus*, *Nocardiopsis*, *Natribacillus*, *Solibacillus*, *Geomicrobium*, *Salinicoccus* and *Glutamicibacter* were positively correlated with 4-oxoisophorone and 1,4-cyclohexanedione, 2,2,6-trimethyl-, which could increase the sweet, hay, nutty, and woody aroma.

**Figure 9:**
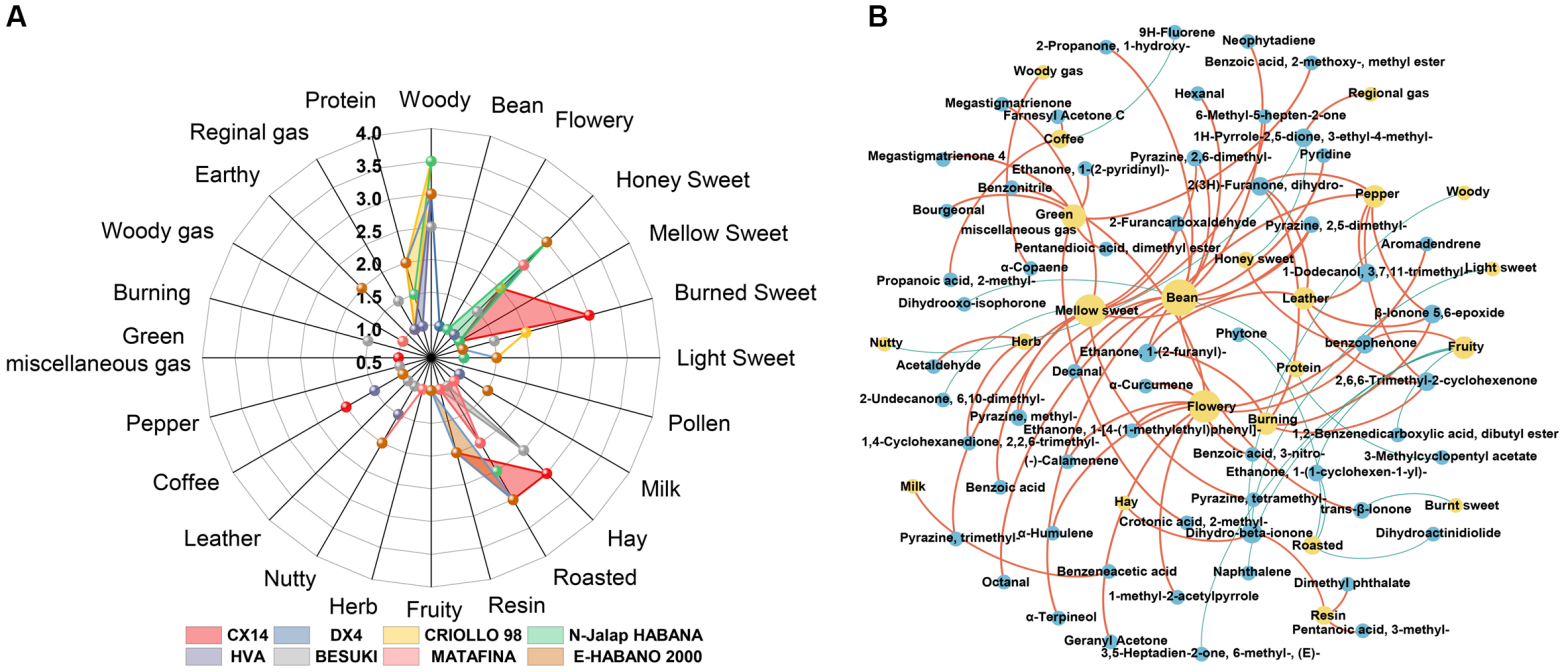
Aroma composition of CTLs and interaction network between VFCs and aroma. **(A)** Radar chart of the aroma from different varieties of CTLs. **(B)** Interaction network between the VFCs and aroma. Red represents positive correlation and blue represents negative correlation.

Radar charts were used to identify the aroma of different CTL varieties at the end of stacking fermentation. The results are shown in Figure 9A, where the woody aroma was found to be the most prominent among the different CTL varieties, with each sample being supplemented with another aroma. Burned sweet aroma was the most prominent aroma in CX14 CTLs, supplemented by hay and roasting aromas. DX4 CTLs had the highest scores in woody and mellow sweet, supplemented by resin aroma and nutty flavor. CRIOLLO 98 had the highest scores in woody, supplemented by mellow sweet, resin and bean aroma. N-Jalap HABANA with mainly woody aroma, supplemented by baking and honey sweetness. HVA had the highest scores in woody, supplemented by roasting, coffee and nutty aroma; BESUKI had the highest scores in woody and hay, supplemented by honey sweet and burned sweet aroma; MATA FINA had the highest scores in woody aroma, supplemented by honey, roasted and nutty aroma. E-HABANO 2000 had the highest scores in woody and honeysweet, complemented by roasted, resin and nutty aromas.

Using Spearman correlation analysis, the interaction network between volatile aroma was established; *ρ* < 0.05 and *r* > 0.6 were defined as significant positive correlations, whilst *ρ* < 0.05 and *r* < −0.6 were defined as significant negative correlations, with the results being shown in Figure 9B. The characteristic VFCs of CTLs showed a stronger positive correlation with many aromas, such as bean aroma, which was positively related to nonanal, decanal, pyrazine, 2,5-dimethyl-, furfural, pyrazine, 2,6-dimethyl-, and α-terpineol. Meanwhile, the coffee aroma was positively correlated with farnesyl acetone C and propanoic acid, 2-methyl-, whilst the flowery aroma was positively correlated with geranyl acetone, benzophenone, benzonitrile, and α-curcumene. Hay aroma was positively correlated with dihydro-beta-ionone; Honey sweet aroma was positively correlated with benzophenone; Mellow sweet aroma was positively correlated with megastigmatrienone, pyrazine, tetramethyl-, and furfural; Pepper aroma was positively correlated with 1-dodecanol, 3,7,11-trimethyl-, 2,6,6-trimethyl-2-cyclohexenone and ethanone, 1-(2-furanyl)-. The resin aroma was positively correlated with dimethyl phthalate and dihydro-beta-ionone.

The characteristic VFCs of different CTLs varieties were positively correlated with the aroma of CTLs, such as the characteristic VFCs of DX4 CTLs, including nonanal and pyrazine, tetramethyl, which corresponded to sweet, roasted, and nutty honey aromas, respectively. In N-Jalap HABANA CTLs, geranyl acetone and α-curcumene were positively correlated with the honey sweet aroma. The characteristic VFCs farnesyl acetone C and propanoic acid, 2-methyl-, were positively correlated with coffee and roasted aroma in HVA CTLs. In BESUKI CTLs, dihydro-beta-ionone was positively correlated with hay aroma; In MATA FINA CTLs, acetaldehyde and 4-oxoisophorone were positively correlated with honey sweet and roasted aroma, respectively. In CX14 CTLs, characteristic VFCs were not strongly correlated with the aroma, but the VFCs with a pleasant aroma, such as the characteristic VFCs ethanone, 1-(2-pyridinyl)-, tigelic acid, benzoic acid, 2-methoxy-, methyl ester, and bourgeonal, corresponded to the burn sweet, roasted and hay aromas, respectively. Furthermore, the higher contents of ethanone, 1-(3-pyridinyl)-and pentanoic acid, 3-methyl- in E-HABANO 2000 CTLs were positively correlated with featured roasted and resin aromas, respectively. The above results showed that the characteristic VFCs of CTLs had an important contribution to the aroma.

## 4. Discussion

**Figure 10:**
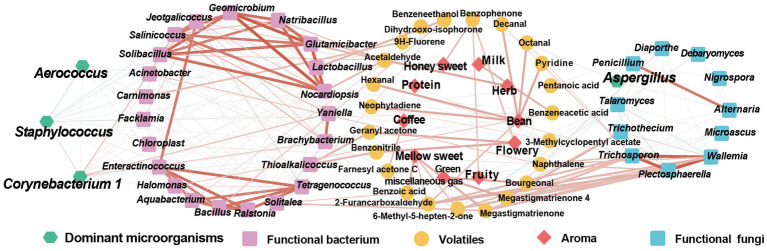
Interaction network among microorganism-VFCs-aroma. Red represents positive correlation, blue represents negative correlation.

CTLs that go through stacking fermentation could better improve their aroma quality, aroma amount, and mellowness, whilst decreasing irritation; therefore, it is important to study the influence of the microbial community on aroma changes and the mechanism of this. In this study, through the analysis of the microbial community composition and VFCs of CTLs, the interaction network between microorganisms, volatile and aroma was established, as shown in Figure 10. This which provides theoretical support for the analysis of the mechanism of microorganisms and the changes in VFCs during stacking fermentation as well as the development of cigar stacking fermentation microbial agents.

The results of amplicon sequencing showed that the microbial community structures of different CTLs varieties were quite different during stacking fermentation, whilst the dominant microbial structures remained similar. The dominant bacterial genera here were *Staphylococcus*, *Corynebacterium 1*, *Aerococcus*, and *Aspergillus*, which was similar to the results found previously by Zheng L. L. et al. (2022) and Zheng et al. (2022a). The results of microbial interaction analysis showed that during stacking fermentation, the dominant microorganisms were strongly related to the CTLs characteristic microorganisms, whilst the abundance of characteristic microorganisms was affected by the correlation and abundance of dominant microorganisms. These characteristic microorganisms also showed a positive correlation with the aroma and could be defined as functional microorganisms in the stacking fermentation process. The characteristic bacterial genera *Jeotgalicoccus*, *Geomicrobium*, *Glutamicibacter*, and *Yaniella* were positively correlated with *Corynebacterium 1*. Furthermore, *Carnimonas* and *Yaniella* positively correlated with *Aerococcus*. Among them, *Carnimonas* was positively correlated with Dihydro-beta-ionone; *Yaniella* was positively correlated with nicotine, and *Geomicrobium*, *Salinicoccus*, *Glutamicibacter* were positively correlated with VFCs such as 1,4-cyclohexanedione, 2,2,6-trimethyl-, and 4-oxoisophorone. Among CTLs, *Jeotgalicoccus*, *Glutamicibacter*, *Yaniella*, and other characteristic microorganisms have good salt tolerance, can better adapt to the alkaline environment of CTLs, and have functions including enzyme production and volatile transformation (Mokashe et al., 2015; Xiong et al., 2019; Zhang et al., 2022). Therefore, during the stacking fermentation process, CTL-dominant microorganisms may have affected the succession changes of functional microorganisms and microbial communities through microbial interactions, thereby affecting the transformation and aroma composition of CTLs (Zhou J. et al., 2021; Zheng et al., 2022a). Jia et al. (2023) found that *Candida* only accounted for 0.42% of the total microbial abundance of the community, but it was used as a symbiotic flora in the microbial community and characteristic microorganisms in the late fermentation period. Not only did it degrade nitrogenous compounds, but it also improved cigar flavor and shortened fermentation period.

Stacking fermentation is an important part of mellowing smoke gas and improving tobacco permeability and aroma, with different varieties of CTLs having their own aroma and characteristic VFCs. The analysis of VFCs between raw materials and the end of fermentation showed that VFCs were mainly increased during stacking fermentation, including Maillard reaction products such as nonanal, pyrazine, tetramethyl-, furfural and ethanone, 1-(3-pyridinyl)-. This is in addition to, carotenoid degradation products such as dihydroactinidiolide, geranyl acetone, 2-pentadecanone, 6,10,14-trimethyl-, 2-undecanone, 6,10-dimethyl-, 1,4-cyclohexanedione, 2,2,6-trimethyl-, 6-methyl-3,5-heptandien-2-one, 2,6,6-trimethyl-2-cyclohexenone, as well as nicotine degradation products such as nicotyrine and myosmine. The above results showed the transformation of small molecular substances such as carotenoid degradation, Maillard reaction, and nicotine degradation during the stacking fermentation process, causing the reduction of miscellaneous gas and the improvement of quality and aroma of CTLs (Lin et al., 2016; Popova et al., 2019; Yu H. et al., 2021). Carotenoid degradation products are considered to be an important source of flavor in CTLs, among which geranyl acetone and dihydroactinidiolide have a woody aroma, green flavor, and can mellow, and flue smoke gas whilst increasing the smoke concentration. 2,6,6-trimethyl-2-cyclohexenone, 2-undecanone, 6,10-dimethyl- and 1,4-cyclohexanedione, 2,2,6-trimethyl- usually have sweet and violet flavors (Gupta, 2015). Maillard reaction products are important aromagenic components of CTLs that burn with a sweet and roasted aroma, and studies have shown that microorganisms can synthesize aminoketones, which are the precursors of pyrazines, and further convert them into pyrazines (Zhang et al., 2019; Mortzfeld et al., 2020; Yu H. et al., 2021). pyrazine, tetramethyl-, pyrazine, 2,5-dimethyl-, furfural and pyrazine, 2,6-dimethyl- have a roasted aroma and burn sweet aroma, commonly used as CTLs flavor additives (Gupta, 2015). Compared with other tobacco products, nicotine had relatively low content in CTLs. Due to the dependence, nicotine exposure and subsequently risk of lung, laryngeal and oral cancers, the degradation of nicotine during cigar stacking fermentation helped to reduce the risk (Henningfield et al., 1996; Baker et al., 2000). This may be useful to control the nicotine content and for potential research and regulatory efforts as well as appropriate product for consumers (Koszowski et al., 2018).

Through the correlation analysis of aroma components and tobacco leaf aroma characteristics, it was found that the characteristic aroma components of different varieties of CTLs corresponded to the prominent aroma of tobacco leaves. For example, *Diaporthe* and *Trichothecium* in CX14 CTLs are positively correlated with ethanone, 1-(2-pyridinyl)-, tigelic acid, megastigmatrienone 4, and bourgeonal, which can enhance the flowery and light sweet aroma of CTLs. In DX4 CTLs, *Chloroplast* were positively correlated with ethanone, 1-(1-cyclohexen-1-yl)-, whilst *Trichosporon* was positively correlated with pyrazine, tetramethyl-, which was positively correlated with a roasted and burnt sweet aroma. The above results showed that the characteristic microorganisms from different varieties of CTLs during stacking fermentation might be functional microorganisms enhancing the characteristic aroma of CTLs, which had an important impact on aroma transformation and microbial community interactions. These results have theoretical significance for screening functional strains and producing cigar products with characteristic aromas.

## 5. Conclusion

In this study, multi-omics technology was used to analyze different CTL varieties in the stacking fermentation process. The commonality and difference changes in microbial communities and VFCs during stacking fermentation were investigated, with the correlation between microorganisms and VFCs was revealed. This provides technical support for the optimization of the stacking fermentation process whilst improving the quality of cigars. This study found that the dominant microorganisms in the process of cigar stacking fermentation were *Staphylococcus*, *Corynebacterium 1*, *Aerococcus*, and *Aspergillus*, which mainly affected the composition and community succession of stacking fermentation through microbial interactions, thereby influencing the aroma composition and aroma of CTLs. Characteristic microorganisms such as *Jeotgalicoccus*, *Geomicrobium*, *Glutamicibacter*, and *Yaniella* had strong correlation with dominant microorganisms. Meanwhile, characteristic microorganisms were probably the functional microorganisms with commensal and aroma-producing ability during stacking fermentation. Based on the analysis of VFCs between raw materials and the end of fermentation for different varieties of CTLs, it was found that Maillard reaction products, carotenoid degradation products, and nicotine degradation products were used as the key aroma components for sensory quality improvement and aroma enhancement during the stacking fermentation process. This provides a basis for improving the quality of cigars and the controllability of cigar proportioning in industrial production. In this study, the interaction network analysis of characteristic microorganisms and VFCs was used for the screening of functional microorganisms and the development and application of microbial agents to improve the aroma quality and characteristic aromatic cigar production.

## Data availability statement

The data presented in the study are deposited in the NCBI Sequence Read Archive database, accession number PRJNA937179.

## Author contributions

QW and ZP: conceptualization, data curation, formal analysis, methodology, software, and writing—original drafting. QW, ZP, and LnL: investigation, methodology, and resources. JZ, YP, JW, and LpL: methodology, resources, and project administration. JZ, YP, and JW: funding acquisition, supervision, and writing—reviewing and editing. All authors contributed to the article and approved the submitted version.

## Funding

This study was supported by the China National Tobacco Technology Development Corporation Project (2021JCYL3SX2B011).

## Conflict of interest

YP, LpL, LnL, and JW were employed by China Tobacco Hubei Industrial Co., Ltd, Wuhan, China.

The authors declare that this study received funding from China Tobacco Hubei Industrial Co., Ltd. The funder had the following involvement in the study: investigation, resources, project administration and funding acquisition.

## Publisher’s note

All claims expressed in this article are solely those of the authors and do not necessarily represent those of their affiliated organizations, or those of the publisher, the editors and the reviewers. Any product that may be evaluated in this article, or claim that may be made by its manufacturer, is not guaranteed or endorsed by the publisher.
