# Supplementary material for: Interaction analysis of tobacco leaf microbial community structure and volatiles flavor compounds during cigar stacking fermentation
Source: Front Microbiol. 2023 Aug 10;14:1168122. doi: 10.3389/fmicb.2023.1168122 (PMC10457113; doi:10.3389/fmicb.2023.1168122)
Supplement: Supplementary file 1 [file Data_Sheet_1.docx]

**Supplementary Material**

**Interaction analysis of tobacco leaf microbial community structure and volatiles flavor compounds during cigar stacking fermentation**

Qiaoyin Wu^a,b,c,1^, Zheng Peng^a,c,1^, Yong Pan^d^, Liping Liu^d^, Linlin Li^d^, Juan Zhang^a,b,c^*, Jian Wang^d*^

^a^Key Laboratory of Industrial Biotechnology, Ministry of Education, Jiangnan University, Wuxi 214122, Jiangsu, China;

^b^School of Biotechnology, Jiangnan University, Wuxi 214122, China;

^c^Science Center for Future Foods, Jiangnan University, Wuxi 214122, China;

^d^China Tobacco Hubei Industry Co., Ltd, Wuhan 430040, China;

*Corresponding author: Juan Zhang, Jian Wang

^1^Both authors contributed equally to this work.

*Corresponding author: Jian Wang, Juan Zhang

E-mail addresses: zhangj@jiangnan.edu.cn; wangjian@sx.hbtobacco.cn

1. **Supplementary Tables**

**Supplementary Table 1.** Sample information.

| Sample | Variety | stage | origin |
| --- | --- | --- | --- |
| C0 | CX14 | Raw materiel | Shiyan |
| C1 | CX14 | pre-fermentation | Shiyan |
| C2 | CX14 | mid-fermentation | Shiyan |
| C3 | CX14 | post-fermentation | Shiyan |
| C4 | CX14 | end of fermentation | Shiyan |
| D0 | DX4 | Raw materiel | Deyang |
| D1 | DX4 | pre-fermentation | Deyang |
| D2 | DX4 | mid-fermentation | Deyang |
| D3 | DX4 | post-fermentation | Deyang |
| D4 | DX4 | end of fermentation | Deyang |
| R0 | CRIOLLO 98 | Raw materiel | Dominica |
| R1 | CRIOLLO 98 | pre-fermentation | Dominica |
| R2 | CRIOLLO 98 | mid-fermentation | Dominica |
| R3 | CRIOLLO 98 | post-fermentation | Dominica |
| R4 | CRIOLLO 98 | end of fermentation | Dominica |
| N0 | N-Jalap HABANA | Raw materiel | Dominica |
| N1 | N-Jalap HABANA | pre-fermentation | Dominica |
| N2 | N-Jalap HABANA | mid-fermentation | Dominica |
| N3 | N-Jalap HABANA | post-fermentation | Dominica |
| N4 | N-Jalap HABANA | end of fermentation | Dominica |
| H0 | HVA | Raw materiel | Dominica |
| H1 | HVA | pre-fermentation | Dominica |
| H2 | HVA | mid-fermentation | Dominica |
| H3 | HVA | post-fermentation | Dominica |
| H4 | HVA | end of fermentation | Dominica |
| B0 | BESUKI | Raw materiel | Indonesia |
| B1 | BESUKI | pre-fermentation | Indonesia |
| B2 | BESUKI | mid-fermentation | Indonesia |
| B3 | BESUKI | post-fermentation | Indonesia |
| B4 | BESUKI | end of fermentation | Indonesia |
| M0 | MATA FINA | Raw materiel | Ecuador |
| M1 | MATA FINA | pre-fermentation | Ecuador |
| M2 | MATA FINA | mid-fermentation | Ecuador |
| M3 | MATA FINA | post-fermentation | Ecuador |
| M4 | MATA FINA | end of fermentation | Ecuador |
| E0 | E-HABANO 2000 | Raw materiel | Brazil |
| E1 | E-HABANO 2000 | pre-fermentation | Brazil |
| E2 | E-HABANO 2000 | mid-fermentation | Brazil |
| E3 | E-HABANO 2000 | post-fermentation | Brazil |
| E4 | E-HABANO 2000 | end of fermentation | Brazil |

**Supplementary Table 2.** VIP value of characteristic volatile flavor compounds at the end of fermentation

| Characteristic volatiles | VIP value | Characteristic volatiles | VIP value |
| --- | --- | --- | --- |
| Myosmine | 1.57405 | Benzoic acid, 2-methoxy-, methyl ester | 1.18162 |
| 1,4-Cyclohexanedione, 2,2,6-trimethyl | 1.45416 | 1,2-Benzenedicarboxylic acid, dibutyl ester | 1.15964 |
| Acetaldehyde | 1.3772 | 4-Oxoisophorone | 1.15846 |
| Nonanal | 1.3114 | Ethanone, 1-(1-cyclohexen-1-yl)- | 1.12645 |
| 9H-Fluorene | 1.28166 | 1,2-Benzenedicarboxylic acid, dimethyl ester | 1.10581 |
| Nicotyrine | 1.28049 | Dihydrooxo isophorone | 1.08895 |
| Pyrazine, tetramethyl- | 1.26478 | Farnesyl Acetone C | 1.07567 |
| 1-Dodecanol, 3,7,11-trimethyl- | 1.25785 | 2-Undecanone, 6,10-dimethyl- | 1.06718 |
| 2,6,6-Trimethyl-2-cyclohexenone | 1.25185 | Pentanoic acid, 3-methyl- | 1.05227 |
| Dihydro-beta-ionone | 1.23125 | Butanoic acid | 1.05106 |
| Acetic Acid | 1.20595 | Geranyl acetone | 1.04046 |
| Ethanone, 1-(2-pyridinyl)- | 1.20081 | Megastigmatrienone 4 | 1.03334 |
| Phytol | 1.19823 | α-Copaene | 1.00776 |
| Crotonic acid, 2-methyl- | 1.19381 | Ethanone, 1-(2-furanyl)- | 1.00584 |
| Propanoic acid, 2-methyl- | 1.02257 | α-Curcumene | 1.00367 |
| Bourgeonal | 1.008 | Isophorone | 1.0079 |

**Supplementary Table 3.** Concentration of volatile flavor compounds

| Compounds | C0 | C4 | D0 | D4 | R0 | R4 | N0 | N4 | H0 | H4 | B0 | B4 | M0 | M4 | E0 | E4 |
| --- | --- | --- | --- | --- | --- | --- | --- | --- | --- | --- | --- | --- | --- | --- | --- | --- |
| Nicotyrine | 0.268223576 | 0.354080121 | 0.210094599 | 0.383091965 | 0.266591619 | 0.438440025 | 0.409386388 | 0.254397026 | 0.349869083 | 0.213048443 | 0.251646339 | 0.357684953 | 0.356756744 | 0.544210517 | 0.343950979 | 0.364948245 |
| Acetic Acid | 0.206217158 | 0.057974906 | 0.26202714 | 0.051831028 | 0.015019085 | 0 | 0.01406443 | 0.204638831 | 0.023274263 | 0 | 0.11304336 | 0.008116465 | 0.125793686 | 0.00154664 | 0.021759509 | 0.147803449 |
| Neophytadiene | 0.063274897 | 0 | 0 | 0.001061803 | 0 | 0 | 0 | 0 | 0 | 0 | 0 | 0 | 0 | 0 | 0.00034211 | 0 |
| Ethanone, 1-(3-Pyridinyl)- | 0.185363637 | 0.321095076 | 0.205534665 | 0.169361645 | 0.417907783 | 0.291374418 | 0.208330042 | 0.260990372 | 0 | 0 | 0.183755579 | 0.382899702 | 0.246511431 | 0.185658773 | 0.256768004 | 0.333679059 |
| Pentanoic Acid, 3-Methyl- | 0.035352805 | 0.020429883 | 0.007936684 | 0 | 0.019300309 | 0 | 0 | 0 | 0.073919501 | 0.106655689 | 0.146824173 | 0.014826568 | 0.015701407 | 0 | 0.025686281 | 0.017300035 |
| Pentanoic Acid | 0.034149687 | 0.019441508 | 0.012932567 | 0 | 0 | 0 | 0 | 0.013714861 | 0.014966987 | 0.072596305 | 0.038133866 | 0.002008382 | 0.003432176 | 0 | 0 | 0.00671792 |
| Myosmine | 0.040822698 | 0.064541308 | 0.036294172 | 0.05327108 | 0.058424068 | 0.125540769 | 0.087230917 | 0.077808973 | 0.072003025 | 0.044481929 | 0.054710857 | 0.059847043 | 0.032383911 | 0.036611027 | 0.048407052 | 0.047791644 |
| (E)-Solanone | 0.029102422 | 0.050949625 | 0.07216098 | 0.073786622 | 0.059074095 | 0.036511952 | 0.049277299 | 0.041541542 | 0.034516566 | 0.039740845 | 0.030387116 | 0.028278537 | 0.019084666 | 0.017960946 | 0.049933144 | 0.016336861 |
| Geranyl Acetone | 0.024205618 | 0 | 0.016503523 | 0 | 0 | 0 | 0 | 0.020456304 | 0 | 0 | 0.011881408 | 0 | 0 | 0 | 0 | 0 |
| Dihydroactindiolide | 0.016245543 | 0.014210271 | 0.021415348 | 0.04352756 | 0.053038778 | 0.029289182 | 0.02450642 | 0.01674937 | 0.061565815 | 0.035451708 | 0.055478991 | 0.028522501 | 0.042160909 | 0.030997431 | 0.065001816 | 0.016041401 |
| Benzoic Acid, Methyl Ester | 0.008590181 | 0 | 0 | 0 | 0 | 0 | 0 | 0 | 0 | 0 | 0 | 0 | 0 | 0 | 0 | 0 |
| (+/-)-p-Menthan-3-ol | 0.008776273 | 0.00137329 | 0.010037482 | 0.003182484 | 0.007072291 | 0.001430797 | 0.003684054 | 0.001758393 | 0.015256391 | 0.003152532 | 0.004455574 | 0 | 0.002977169 | 0.000432238 | 0.009760594 | 0 |
| Benzeneethanol | 0.008344354 | 0 | 0.010386404 | 0 | 0 | 0 | 0 | 0 | 0.002155793 | 0.002978751 | 0 | 0 | 0 | 0 | 0 | 0 |
| Naphthalene | 0.005067127 | 0.006795655 | 0.003182249 | 0.007847225 | 0.002203703 | 0 | 0.006126028 | 0 | 0.002144132 | 0.00178187 | 0.003719818 | 0.001454924 | 0.003760916 | 0.001691414 | 0.010627772 | 0.000620765 |
| Indole | 0.004696429 | 0.001212091 | 0.004674706 | 0.005404988 | 0.005187542 | 0.00244759 | 0.003353749 | 0.002984844 | 0.005432015 | 0.003668068 | 0.000623476 | 0.001026238 | 0.002767228 | 0 | 0.007424482 | 0.000969194 |
| Megastigmatrienone | 0.005050408 | 0.002859759 | 0.009149792 | 0.013075589 | 0 | 0 | 0.003475182 | 0.000953495 | 0 | 0 | 0.001221318 | 0.000302012 | 0.00149778 | 0 | 0.000804527 | 0 |
| Benzoic Acid | 0.003968143 | 0.000775116 | 0 | 0.002414273 | 0.002053112 | 0 | 0 | 0.001246064 | 0 | 0 | 0 | 0 | 0.000767262 | 0 | 0.008028798 | 0 |
| 2-Pentadecanone, 6,10,14-Trimethyl- | 0.004005748 | 0.006832766 | 0.012338449 | 0.018881 | 0.010656485 | 0.008027121 | 0.013853196 | 0.005999392 | 0.018682749 | 0.008368723 | 0.009066619 | 0.008678497 | 0.018977078 | 0.006825554 | 0.015563334 | 0.005445582 |
| Nonanal | 0.001886863 | 0.004470421 | 0.002866353 | 0.005565674 | 0.015305855 | 0.001828554 | 0.007025871 | 0.002764228 | 0.003296744 | 0.001228109 | 0.014674831 | 0.002643749 | 0.005308483 | 0.008314358 | 0.004378666 | 0.000887517 |
| Propanoic Acid | 0.003053846 | 0 | 0.001784799 | 0 | 0 | 0 | 0 | 0 | 0.000368527 | 0.002877983 | 0.003409582 | 0 | 0.001548299 | 0 | 0 | 0.00042599 |
| 1,2-Benzenedicarboxylic Acid, Dibutyl Ester | 0.002691579 | 0.004835575 | 0 | 0.00739438 | 0 | 0.000925115 | 0.002669235 | 0.002151848 | 0 | 0.001754106 | 0.001503901 | 0.001148232 | 0.001261159 | 0.0088277 | 0.01215694 | 0 |
| Benzeneacetic Acid | 0.002071546 | 0 | 0.002349404 | 0.002753433 | 0.001322567 | 0 | 0 | 0.000288053 | 0 | 0.001046794 | 0 | 0 | 0 | 0 | 0.004213527 | 0.000167415 |
| 2-Epi-Α-Funebrene | 0.002043887 | 0 | 0.003402499 | 0 | 0.002468888 | 0 | 0 | 0 | 0.007016279 | 0.001193028 | 0.001707285 | 0 | 0.001296545 | 0 | 0.002598623 | 0 |
| Benzaldehyde | 0.002351774 | 0.002042216 | 0.002658365 | 0 | 0.001090972 | 0.001687492 | 0.004436689 | 0.003991206 | 0.001382558 | 0.000714342 | 0.006717383 | 0.001685277 | 0.002941765 | 0.000993321 | 0.004401538 | 0.001214613 |
| Ethanone, 1-Phenyl- | 0.001908582 | 0.003175255 | 0 | 0.003538632 | 0 | 0.00111467 | 0.004782351 | 0 | 0.00101768 | 0.000448114 | 0.000968453 | 0 | 0.002689969 | 0.000654489 | 0.00668649 | 0 |
| Dihydro-beta-ionone | 0.001150094 | 0.001823343 | 0 | 0 | 0 | 0 | 0.001559819 | 0 | 0 | 0.001176502 | 0.003293088 | 0.002745801 | 0.001767031 | 0.001457887 | 0.001308068 | 0.000469654 |
| 1,2-Benzenedicarboxylic Acid, Dimethyl Ester | 0.001417722 | 0.001136565 | 0 | 0 | 0.000834523 | 0.000391136 | 0 | 0 | 0.001718949 | 0.000412255 | 0 | 0 | 0.001211907 | 0.000436117 | 0.001561652 | 0.000400513 |
| 4-Ketoisophorone | 0.000955299 | 0.000609291 | 0 | 0 | 0 | 0 | 0 | 0 | 0.001054128 | 0 | 0.001809965 | 0 | 0.001366294 | 0.001284837 | 0 | 0.000923848 |
| 3,5-Heptadien-2-One, 6-Methyl-, (E)- | 0.000602356 | 0.001073635 | 0.001189882 | 0.002598514 | 0.000969396 | 0 | 0 | 0 | 0.001713153 | 0.000611258 | 0 | 0.000707803 | 0.001279108 | 0.000319489 | 0.001801023 | 0 |
| Pyrazine, 2,5-Dimethyl- | 0.000537455 | 0.003032626 | 0.000703139 | 0.020558868 | 0.000537961 | 0 | 0.001795326 | 0 | 0.0004793 | 0 | 0 | 0 | 0 | 0 | 0.00235184 | 0 |
| Pyrazine, Tetramethyl- | 0.000456047 | 0.000822855 | 0.000881297 | 0.001008253 | 0.00071566 | 0 | 0.00128447 | 0.00057921 | 0.001217297 | 0.000407558 | 0.001162252 | 0.000524077 | 0.001203512 | 0.000488411 | 0.001171051 | 0.000276407 |
| Dihydrooxo-Isophorone | 0.000286304 | 0.000244637 | 0 | 0 | 0.000309002 | 0 | 0.000543873 | 0.000468228 | 0 | 0.000216307 | 0 | 0.000382189 | 0 | 0 | 0.000255419 | 0 |
| 2-Furancarboxaldehyde | 0.000182475 | 0.000431436 | 0 | 0.000491436 | 0 | 0 | 0 | 0 | 0 | 0 | 0 | 0 | 0.000505902 | 0 | 0.000342112 | 0 |
| 6-Methyl-5-Hepten-2-One | 0.00028288 | 0.000726726 | 0.000372178 | 0.001174249 | 0.00056067 | 0.000330127 | 0.000765605 | 0.000341327 | 0.001024702 | 0.000290905 | 0.00099633 | 0 | 0.001555574 | 0 | 0.002128177 | 0 |
| Ethanone, 1-(1-Cyclohexen-1-Yl)- | 0.000233335 | 0 | 0.000581037 | 0.000556142 | 0.00035987 | 0 | 0.000190299 | 0 | 0.001680788 | 0.000576522 | 0 | 0.000673959 | 0.000525252 | 0.000364691 | 0.000373618 | 0.000228768 |
| Trans-Β-Ionon-5,6-Epoxide | 0.000175064 | 0 | 0.024661113 | 0 | 0 | 0 | 0.053918171 | 0 | 0.033830017 | 0 | 0.019471224 | 0.034071278 | 0 | 0 | 0 | 0 |
| Hexanal | 0.00010347 | 0 | 0 | 0.000471397 | 0.000186058 | 0 | 0.00054524 | 0 | 0.000251143 | 0 | 0 | 0 | 0.000367147 | 0 | 0.000639306 | 0 |
| Α-Copaene | 0.000116692 | 0.000362385 | 0.000303128 | 0 | 0.000476927 | 0 | 0.017344963 | 0.00764715 | 0.000729566 | 0 | 0.000345941 | 0 | 0.000550008 | 0.000362591 | 0.000398833 | 8.16E-05 |
| Decanal | 0 | 0 | 0 | 0.003156815 | 0 | 0 | 0 | 0 | 0.000819113 | 0 | 0.000286925 | 0 | 0.000949884 | 0 | 0.000865041 | 0 |
| Megastigmatrienone 4 | 0 | 0.002158132 | 0 | 0 | 0 | 0 | 0 | 0 | 0 | 0 | 0 | 0 | 0.001099472 | 0 | 0 | 0 |
| 5,9-Undecadien-2-one, 6,10-dimethyl-, (E)- | 0 | 0.029670146 | 0.025815717 | 0.017496254 | 0.030737612 | 0.042766885 | 0.025612866 | 0.020846687 | 0.146746478 | 0.058195136 | 0 | 0 | 0.06057979 | 0.044514538 | 0.024962022 | 0.026344256 |
| Benzoic Acid, 2-Methoxy-, Methyl Ester | 0.006983228 | 0.001004952 | 0 | 0 | 0 | 0 | 0 | 0 | 0 | 0 | 0 | 0 | 0 | 0 | 0 | 0 |
| Benzaldehyde, 3-Hydroxy-4-Methoxy- | 0.000338981 | 0 | 0 | 0 | 0 | 0 | 0 | 0 | 0 | 0 | 0 | 0 | 0 | 0 | 0 | 0 |
| Dihydro Oxoisophorone | 0 | 0 | 0.002647036 | 0 | 0 | 0 | 0 | 0 | 0 | 0 | 0 | 0 | 0 | 0.000912556 | 0 | 0 |
| Β-Ionone | 0 | 0.001617424 | 0 | 0.00315781 | 0 | 0 | 0.00220213 | 0 | 0 | 0 | 0 | 0 | 0.001705888 | 0.002503092 | 0 | 0 |
| 2(3H)-Furanone, Dihydro- | 0.000256623 | 0 | 0.000554407 | 0 | 0 | 0 | 0 | 0 | 0 | 0 | 0 | 0.000229853 | 0 | 0 | 0.000528245 | 0 |
| Farnesyl Acetone C | 0 | 0.004004224 | 0 | 0 | 0 | 0 | 0 | 0 | 0 | 0.005483106 | 0 | 0 | 0 | 0 | 0 | 0 |
| Bourgeonal | 0 | 9.45E-05 | 0 | 0 | 0 | 0 | 0 | 0 | 0 | 0 | 0 | 0 | 0 | 0 | 0 | 0 |
| Pyridine | 0 | 0 | 0 | 0.00213673 | 0 | 0 | 0 | 0 | 0 | 0 | 0 | 0 | 0 | 0 | 0 | 0 |
| Pyrazine, Methyl- | 0 | 0.000374209 | 0 | 0.008285332 | 0 | 0 | 0.001716825 | 0 | 0 | 0 | 0 | 0 | 0 | 0 | 0.001836549 | 0 |
| Octanal | 0 | 0 | 0 | 0.00022235 | 0 | 0 | 0 | 0 | 0 | 0 | 0 | 0 | 0.000100842 | 0 | 0 | 0 |
| 2-Propanone, 1-Hydroxy- | 0 | 0 | 0 | 0.004470615 | 0 | 0 | 0 | 0 | 0 | 0 | 0 | 0 | 0 | 0 | 0 | 0 |
| Pyrazine, 2,6-Dimethyl- | 0 | 0.00172962 | 0 | 0.022945486 | 0.000827163 | 0 | 0 | 0 | 0 | 0 | 0 | 0 | 0 | 0 | 0.002447634 | 0 |
| Pyrazine, Trimethyl- | 0 | 0 | 0.000160866 | 0.002135459 | 0.000310363 | 0 | 0.003319954 | 0.000840896 | 0 | 0 | 0 | 0 | 0 | 0 | 0 | 0 |
| Benzeneacetaldehyde | 0 | 0 | 0.007091982 | 0 | 0 | 0 | 0 | 0 | 0 | 0 | 0.001590817 | 0 | 0 | 0 | 0 | 0 |
| α-Terpineol | 0 | 0 | 0 | 0.002551476 | 0 | 0 | 0 | 0 | 0 | 0 | 0 | 0 | 0 | 0 | 0 | 0 |
| Cis-3-Hexenal | 0 | 0 | 0 | 0 | 0 | 0 | 0 | 0 | 0 | 0 | 0 | 0 | 0.000108816 | 0 | 0 | 0 |
| Benzophenone | 0 | 0 | 0.001281787 | 0 | 0.000773296 | 0 | 0 | 0.001546195 | 0 | 0 | 0 | 0 | 0 | 0 | 0 | 0.000406001 |
| Β-Damascenone | 0 | 0 | 0.001354999 | 0 | 0 | 0 | 0 | 0 | 0 | 0 | 0 | 0 | 0 | 0 | 0 | 0 |
| 2,6,6-Trimethyl-2-Cyclohexenone | 0 | 0 | 0.0020238 | 0 | 0.000644302 | 0 | 0 | 0 | 0 | 0 | 0 | 0.001606577 | 0 | 0 | 0 | 0.000384138 |
| Phytol | 0 | 0 | 0.000354869 | 0 | 0.000253566 | 0.000168522 | 0.000287835 | 0 | 0.000667519 | 0 | 0.000982593 | 0.00118185 | 0.001156377 | 0 | 0.000275548 | 0.00016117 |
| Β-Elemene | 0 | 0 | 0 | 0 | 0 | 0 | 0.001349314 | 0 | 0.000605225 | 0 | 0 | 0 | 0 | 0 | 0 | 0 |
| Widdrene | 0 | 0 | 0.000377163 | 0 | 0 | 0 | 0 | 0 | 0 | 0 | 0 | 0 | 0 | 0 | 0 | 0 |
| 4-Oxoisophorone | 0 | 0 | 0 | 0 | 0.000602049 | 0 | 0 | 0 | 0 | 0 | 0 | 0 | 0 | 0 | 0 | 0 |
| Propanoic Acid, 2-Methyl- | 0 | 0.0032606 | 0 | 0 | 0 | 0 | 0 | 0 | 0 | 0.002886327 | 0 | 0 | 0 | 0 | 0 | 0 |
| Lavander lactone | 0 | 0 | 0 | 0 | 0 | 0 | 0 | 0 | 0.002706233 | 0 | 0 | 0 | 0 | 0 | 0 | 0 |
| 2-Undecanone, 6,10-Dimethyl- | 0 | 0 | 0 | 0 | 0.003334417 | 0.00526116 | 0.003577625 | 0.002310149 | 0.007685679 | 0.008082831 | 0.003099567 | 0.006605897 | 0.010111538 | 0.011076195 | 0.006992722 | 0.004971135 |
| Γ-Muurolene | 0 | 0 | 0.001248086 | 0 | 0 | 0 | 0.002076279 | 0 | 0.001796953 | 0 | 0 | 0 | 0 | 0 | 0 | 0 |
| Pyrazine, 2,3-Dimethyl- | 0 | 0 | 0 | 0 | 0 | 0 | 0.000376507 | 0 | 0 | 0 | 0.000228689 | 0 | 0 | 0 | 0 | 0 |
| Α-Humulene | 0 | 0 | 0 | 0 | 0 | 0 | 0.004120837 | 0.001294431 | 0 | 0 | 0 | 0 | 0 | 0 | 0 | 0 |
| Aromadendrene | 0 | 0 | 0 | 0 | 0 | 0 | 0.001526141 | 0.000743828 | 0 | 0 | 0 | 0 | 0 | 0 | 0 | 0 |
| 1S,Cis-Calamenene | 0 | 0 | 0 | 0 | 0 | 0 | 0.00703273 | 0.005334922 | 0 | 0 | 0 | 0 | 0 | 0 | 0 | 0 |
| 4'-Isopropylacetophenone | 0 | 0 | 0 | 0 | 0 | 0 | 0 | 0.000258005 | 0 | 0 | 0 | 0 | 0 | 0 | 0 | 0 |
| 2-Hexenal | 0 | 0 | 0 | 0 | 0 | 0 | 0 | 0 | 0 | 0 | 0 | 0 | 0 | 0 | 0.000302694 | 0 |
| Ethanone, 1-(2-Pyridinyl)- | 0 | 0.000122413 | 0 | 0 | 0 | 0 | 5.10E-05 | 0 | 0 | 0 | 5.71E-05 | 0 | 0 | 0 | 0.000131983 | 0 |
| Ethanol, 2-(2-Ethoxyethoxy)- | 0.000442948 | 0.000532886 | 0.001458569 | 0 | 0.00129014 | 0.000918494 | 0 | 0.001141613 | 0.001273962 | 0.000864877 | 0.001302595 | 0.000869383 | 0.001008886 | 0.000974406 | 0.001331824 | 0.000643476 |
| 1-Dodecanol, 3,7,11-Trimethyl- | 0 | 0 | 0 | 0 | 0 | 0 | 0 | 0 | 0.002196708 | 0 | 0.000474017 | 0.001104021 | 0.000658701 | 0 | 0.001636185 | 0 |
| Trans-Β-Ionone | 0 | 0 | 0 | 0.00315781 | 0 | 0 | 0 | 0 | 0 | 0 | 0 | 0 | 0 | 0.001365344 | 0 | 0 |
| 1-Heptanol | 0 | 0 | 0 | 0 | 0 | 0 | 0 | 0 | 0 | 0 | 0 | 0 | 0 | 0 | 0.000332297 | 0 |
| Ethanone, 1-(1-Methyl-1H-Pyrrol-2-Yl)- | 0 | 0 | 0 | 0 | 0 | 0 | 0 | 0.000560193 | 0 | 0 | 0 | 0 | 0 | 0 | 0.000704935 | 0 |
| 9H-Fluorene | 0 | 0 | 0 | 0 | 0 | 0 | 0 | 5.27E-05 | 0 | 0 | 0.000372952 | 0.000358152 | 0.0001868 | 9.11E-05 | 0 | 0 |
| Benzonitrile | 0 | 0 | 0 | 0 | 0 | 0 | 0 | 0.000351498 | 0 | 0 | 0 | 0 | 0 | 0 | 0 | 0 |
| Acetaldehyde | 0 | 0 | 0 | 0 | 0 | 0 | 0 | 0 | 0 | 0 | 0 | 0 | 0 | 0.000740164 | 0 | 0 |
| Isooctopherone | 0 | 0 | 0 | 0 | 0 | 0 | 0 | 0 | 0 | 0.000303981 | 0 | 0 | 0 | 0 | 0 | 0 |
| Crotonic Acid, 2-Methyl- | 0 | 0.001404302 | 0 | 0 | 0 | 0 | 0 | 0 | 0 | 0 | 0 | 0 | 0 | 0 | 0 | 0 |
| Dl-Limonene | 0 | 0 | 0 | 0 | 0 | 0 | 0 | 0 | 0 | 0 | 0 | 0 | 0.00013299 | 0 | 0.000112304 | 0 |
| Pentanedioic Acid, Dimethyl Ester | 0 | 2.88E-05 | 0 | 0 | 0 | 0 | 0 | 0 | 0 | 0 | 0 | 0 | 0 | 0 | 0 | 0 |
| Benzoic Acid, 3-Nitro- | 0 | 0 | 0 | 0 | 0 | 0 | 0.000237996 | 0.000233502 | 0 | 0 | 0 | 0 | 0 | 0 | 0 | 0 |
| Ethanone, 1-(2-Furanyl)- | 0 | 0 | 0 | 0 | 0 | 0 | 0 | 0 | 0 | 0 | 0 | 0.001052915 | 0 | 0 | 0 | 0 |
| α-curcumene | 0 | 0 | 0 | 0 | 0 | 0 | 0.004511936 | 0.002580553 | 0 | 0 | 0 | 0 | 0.001407772 | 0 | 0 | 0 |
| Butanoic Acid | 0 | 0 | 0 | 0 | 0 | 0 | 0 | 0 | 0 | 0.00191035 | 0 | 0 | 0 | 0 | 0 | 0 |
| 2,3-Butanedione | 0 | 0 | 0 | 0 | 0 | 0 | 0.000961839 | 0 | 0 | 0 | 0 | 0 | 0 | 0 | 0 | 0 |
